# On the brink of isolation: Population estimates of the Araguaian river dolphin in a human-impacted region in Brazil

**DOI:** 10.1371/journal.pone.0231224

**Published:** 2020-04-22

**Authors:** Mariana Paschoalini, Rafael Marques Almeida, Fernando Trujillo, Gabriel Melo-Santos, Miriam Marmontel, Heloíse Julião Pavanato, Federico Mosquera Guerra, Nathali Ristau, Alexandre Novaes Zerbini

**Affiliations:** 1 Laboratório de Ecologia Comportamental e Bioacustica, Programa de Pós-Graduação em Ecologia, Universidade Federal de Juiz de Fora, Juiz de Fora, Minas Gerais, Brazil; 2 Instituto Aqualie, Juiz de Fora, Minas Gerais, Brazil; 3 Grupo de Pesquisa em Mamíferos Aquáticos Amazônicos, Instituto de Desenvolvimento Sustentável Mamirauá, Tefé, Brazil; 4 Department of Ecology and Evolutionary Biology, Postdoctoral Fellow in Sustainability, Atkinson Center for a Sustainable Future, Cornell University, New York, United States of America; 5 Fundación Omacha, Bogotá, Colombia; 6 Sea Mammal Research Unit, Scottish Oceans Institute, School of Biology, University of St Andrews, Saint Andrews, Scotland; 7 BioMA–Biologia e Conservação de Mamíferos Aquáticos Amazônicos, Belém, Brazil; 8 Departament of Mathematic and Statistics, University of Otago, Dunedim, New Zeland; 9 Departamento de Ciências Biológicas, Universidade Federal do Maranhão, Cidade Universitária, São Luís, Maranhão, Brazil; 10 Joint Institute for the Study of Atmosphere and Ocean (JISAO)/University of Washington and Marine Mammal Laboratory, Alaska Fisheries Science Center, NOAA Fisheries, Sand Point Way NE, Seattle, Washington, United States of America; 11 Cascadia Research Collective, Olympia, Washington, United States of America; 12 Marine Ecology and Telemetry Research, Seabeck, Washington, United States of America; National Cheng Kung University, TAIWAN

## Abstract

Populations of freshwater dolphins are declining in response to increased human pressure, including habitat degradation, overfishing, bycatch, poaching and obstruction of free-flowing river corridors by dams. At least three river dolphin species occur in South America: the Amazonian river dolphin, or boto (*Inia geoffrensis*), the Bolivian river dolphin (*Inia boliviensis*) and the tucuxi (*Sotalia fluviatilis*). A fourth species, the Araguaian boto (*Inia araguaiaensis*), been proposed for the Tocantins-Araguaia, a large river basin in northern Brazil. Here we show that the Araguaian boto population in the Tocantins River is relatively small (N = 1083, CV = 0.52). During a survey to estimate density and abundance, 138 groups (198 individuals) of botos were observed along a ~600 km stretch of the Tocantins River in five different habitats (river margin, river channel, channel, island margin, and a dam reservoir). Overall, lower densities of the Araguaian boto were registered downstream of the Tucuruí dam, the world’s fifth largest hydropower dam. Density was 68% lower in the river margin habitat downstream (0.23 ind./km^2^, CV = 0.92) than upstream (0.72 ind./km^2^, CV = 0.53). In addition, density within the Tucuruí reservoir decreases from upstream areas towards the dam. Geographic post-stratification of data into sub-regions (downstream, reservoir, upstream) in relation to the Tucuruí dam helped to reduce CV by ~70%, which illustrates the high variability in the encounter rate in these areas. Our findings suggest that the Araguaian boto population has been impacted by the construction of the Tucuruí dam. The construction of other dams proposed for the Tocantins-Araguaia basin should be planned strategically to minimize overlapping with the Araguaian boto distribution. Coordinated conservation actions are imperative to prevent the Araguaian boto from reaching extinction or near-extinction as some of their Asian counterparts such as the Yangtze, Ganges, and Indus river dolphins.

## Introduction

Freshwater is a driver of development subject to multiple anthropogenic stressors [1]. Riverine ecosystems provide resources for food (e.g., fisheries, irrigation and aquaculture), power generation, transport, and sanitation for human societies [2–4]. River dolphins are a polyphyletic group of cetaceans only found in Northern South America and Southern Asia [5]. Given their habitat, these dolphins are under great human pressure [6, 7]. Regions of overlap between river dolphins and human activities are sites of disturbance and threat for those species [8]. Such threats have direct impact on dolphins or their environment, including habitat degradation, incidental mortality events (e.g., bycatch), poaching, food depletion, and bioaccumulation of heavy metals [9, 3, 8, 10–18]. In addition, population fragmentation in response to the development of hydroelectric dams is of particular concern given the increasing trend in dam planning and construction in countries with emerging economies where river dolphin species commonly occur [19, 20].

Subjected to high population fragmentation by dam constructions, the baiji (Yangtze river dolphin, *Lipotes vexillifer*) was declared “functionally extinct” in China [21]. This is a clear example of the medium- to long-term negative effects of human activities on biodiversity and an alert about the magnitude of the problems involving dams and widely distributed species such as river dolphins. Other freshwater dolphin species such as the Ganges river dolphin (*Platanista gangetica*) and the Irrawaddy dolphin (*Orcaella brevirostris*) are currently classified as Endangered and Vulnerable, respectively, by the International Union for Conservation of Nature (IUCN), due to similar threats [22, 23].

In South America, at least three river dolphin species (*Inia geoffrensis*, *Inia boliviensis* and *Sotalia fluviatilis*) occur in three river basins (Amazon, Orinoco and Tocantins-Araguaia [24–27, 3, 28]). Similarly to Asian river dolphins, the *Inia* species (locally known as boto) were recently classified as Endangered by the IUCN [29]. A third species of *Inia* has been proposed for the Tocantins-Araguaia river basin, *Inia araguaiaensis* (Hrbek, Farias, Dutra & da Silva, 2014), hereafter referred to as Araguaian boto [28]. The Committee on Taxonomy of the Society for Marine Mammalogy [30] has yet to recognize the Araguaian boto as a new species due to the need for additional morphological information to verify the specific status of these dolphins. Thus, in this study, we refer to the Araguaian boto as a population of *Inia*, distinct from botos found in the Amazon River basin.

The Araguaian boto’s habitat has been fragmented since 1984 when the Tucuruí dam, the world’s fifth largest hydropower dam in terms of energy generation capacity, was constructed. This dam is located in the lower-medium Tocantins River and was responsible for important changes in the river’s landscape, limnology, and hydrology [31–33]. While not documented, changes in dolphin distribution and density upstream and downstream are expected as a result of dam construction.

This study presents estimates of density and abundance for the Araguaian boto in the medium and lower Tocantins River in Northern Brazil and advances current understanding of spatial and temporal dynamics of a freshwater cetacean in an impacted and fragmented riverscape. These estimates are also important in a conservation context. New hydroelectric plants planned for the Tocantins-Araguaia River basin [20] will likely further reduce the river network connectivity, disrupt movements of dolphins and fish, and potentially lead to reproductive isolation. Present estimates can therefore be used as a baseline to assess potential population impacts of new dams. We also discuss the potential effects of damming on population fragmentation and river connectivity.

## Material and methods

### Study area

**Fig 1:**
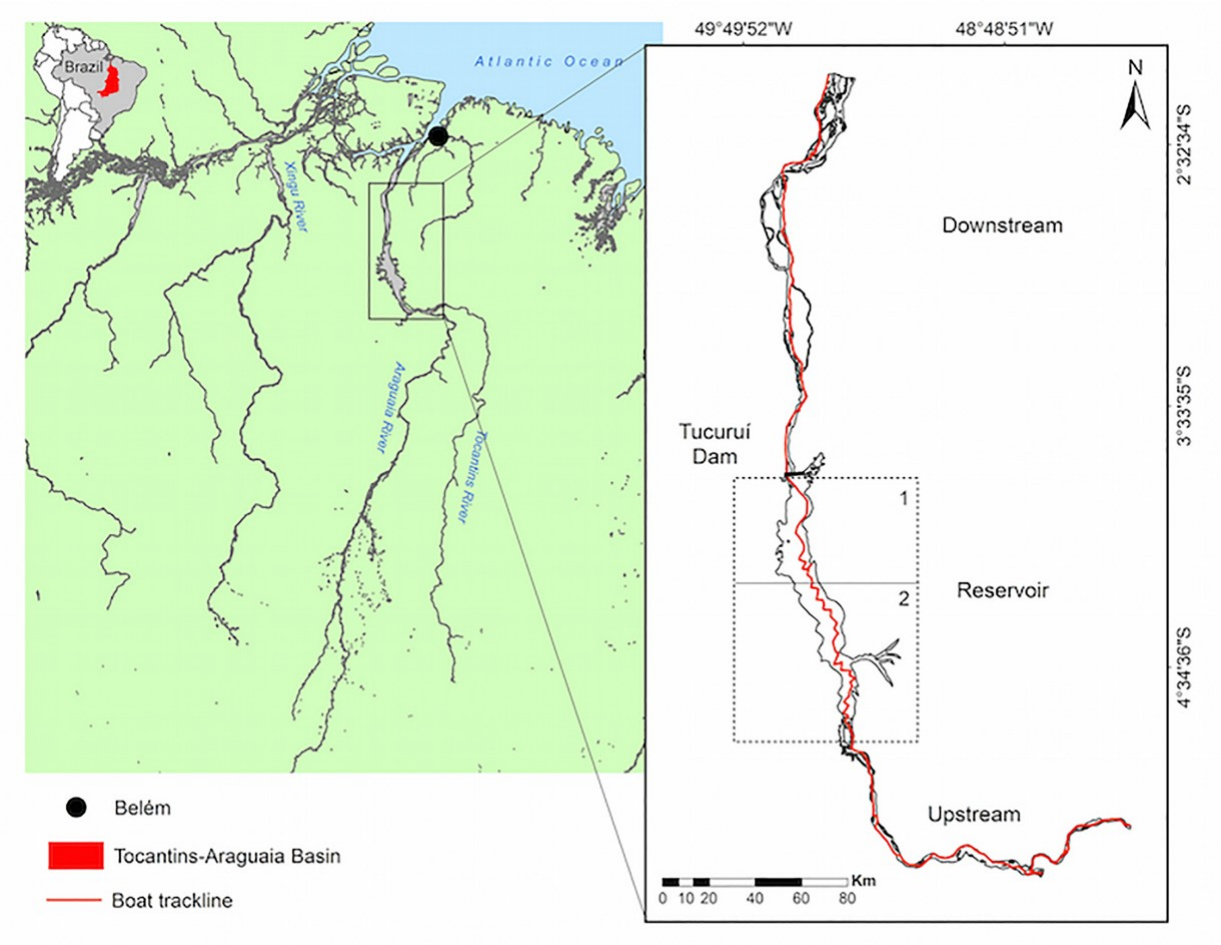
Study area. Map of the surveyed area in the Tocantins River (Tocantins-Araguaia River Basin, Northern Brazil) 2014 survey. The inset shows the realized trackline effort.

The Tocantins-Araguaia River basin, formed by the Tocantins and Araguaia rivers and their tributaries (Fig 1), is the largest hydrographic basin entirely in Brazilian territory. Rivers in this basin flow from an inland region known as the Brazilian Shield into the Atlantic Ocean alongside the Amazon River [9]. The Tocantins-Araguaia basin became disconnected from the Amazon basin during the transition of the Pliocene to the Pleistocene period [28]. The only remaining link between the two basins corresponds to a narrow channel in the Amazon delta where the Tocantins River drains [34]. The Tocantins is the longest clear-water river in Brazil (length ~ 2600 km) and is characteristically deprived of nutrients, ions, and sediments [35, 36]. Rapids and falls are the most common aquatic habitats in the upper course of the Tocantins; they are less common in the middle reaches, and form an important habitat on the medium-lower course, which is now largely inundated by the Tucuruí reservoir [37]. The period of rising water goes from October to April, with high-water peaks in February (upper Tocantins) and March (middle and lower Tocantins) [37, 9]. The lower Tocantins, downstream of the Tucuruí dam, has many marginal lakes and numerous islands; it is influenced by both the annual rise and fall of the main river and by tidal cycles.

### Ethics statement

This study did not make use of human or animal subjects that requires specific permits to research. Scientific expedition to the Tocantins River was conducted within the project "Biologia e ecologia de botos vemelhos" under permits of the Brazilian Institute of the Environment and Renewable Natural Resources (Instituto Brasileiro do Meio Ambiente e dos Recursos Naturais Renováveis, IBAMA, SISBIO (Sistema de Autorização e Informação em Biodiversidade) number 50806) to the Amazon Aquatic Mammals Research Group (IDSM—Instituto de Desenvolvimento Sustentável Mamirauá).

### Survey design and field work

A visual boat-based survey was conducted in the Tocantins River (2° 16’S, 49° 29’W to 5° 11’S, 48° 19’W) from 12 to 19 March 2014, during the high-water level period (Fig 1). Because the Tucuruí dam changed the river’s shape, the survey area was divided into three sub-regions, i.e. strata: (1) downstream of the Tucuruí dam, (2) reservoir (the artificial lake formed by the Tucuruí dam), and (3) upstream of the Tucuruí dam reservoir (Fig 1). The reservoir (artificial lake) was further divided into two sections according to the shape and hydrological features: Reservoir 1: the most altered region by flooding near the dam; Reservoir 2: upstream region where the main channel presents characteristics similar to the natural river channel. The search effort comprised 585.9 km along these three stretches of the Tocantins River, covering an area of 2,657 km^2^ (Table 1).

**Table 1:** River dolphin search effort conducted across the Tocantins River in 2014 by sub-regions (stratum) in relation to the Tucuruí dam.

Stratum / sub-region	*k*	*L* (km)	A (km^2^)
**Downstream**	115	252.6	1169
**Reservoir 1**	17	42.1	331
**Reservoir 2**	43	93.93	342
**Upstream**	97	197.2	385

k, number of transects; L, realized effort; A, calculated area.

A double-decker boat (7 m high and 12 m long) was used as observation platform, and data collection was performed according to the line transect sampling methodology [38] to estimate density and abundance of river dolphins [39–41, 10, 18]. A mixed sampling protocol combining strip transects (survey lines parallel to the margin) and line transects (survey lines crossing the river channel) was implemented to survey the different habitats used by the Araguaian boto [10]. A total of 133 line transects (276.6 km of effort) and 139 strip transects (309.3 km of effort) were surveyed during good and moderate sighting conditions with no rain, low glare, and relatively calm waters (small ripples scale 0–2, which is equivalent to 0–3 in the Beaufort scale). The average length of transects was 2.5 km. Sighting data were collected in passing mode [42] at an average speed of 12 km/h. The survey was designed to maintain a pre-established distance of 100 m from the margin for the strip transects (i.e., a 200 m strip width) (Gomez-Salazar et al. [10]). Due to the presence of rocks and currents along the margins, the distance was often greater.

A team of nine observers searched for river dolphins and rotated every hour between 7-meter-high observation platforms on the bow (Platform 1) and on the stern (Platform 2). On each platform, observers alternated across three positions: port, data recorder and starboard. The port and starboard observers actively searched for dolphins from 10° on the opposite side to 90° on their own side, and a third person was responsible for data recording. After completing the rotation cycle by platform, each researcher rested for at least two hours. The overlap in the observers’ searching fields was designed to minimize the probability of missing animals in the vicinity of the trackline. Data on dolphin sighting was recorded assuming the configuration of ‘one-way’ independency, i.e., the observers on the stern platform were unaware of detections made by those on the bow. This method enables the calculation of key values for capture-recapture models to estimate detection probability (g (0)) on the trackline [43–48].

Observers searched for dolphins with naked eye and used angle boards to measure the radial angle between the sighting and the trackline. For each sighting, the observers recorded the species, group size, presence of calves, radial distance between the sighting and the vessel, the radial angle, distances from the dolphin groups to the margin, habitat type (river margin, river channel–the center of the river, channel, confluence, island margin, lake, tributary) [10], and weather and visibility conditions.

### Data analysis

Analyses were performed using the package *Distance* and *MRDS* in the open-source software R (version 3.4.3, [49]). The population size of tucuxi dolphins (*Sotalia fluvitalis*) could not be assessed due to the low number of sightings (9 groups, n = 17 individuals), and also because they were concentrated in a limited region of the study area. Therefore, hereafter we focus on the results for Araguaian boto. Transects were grouped in two datasets, cross-channel (line transects) and parallel (strip transects) survey lines, and analyzed separately.

Density estimates were computed for each habitat type classified during fieldwork by means of stratification [38, 50], since the density gradient for river dolphins is known to be greater in some river areas [40, 41, 10, 18]. Additionally, geographic post-stratification of the study area into stratum in relation to the Tucuruí dam (downstream, reservoir 1, reservoir 2, upstream) allowed us to investigate the potential effect of the heterogeneity of densities across the study area, which are expected to occur because of substantial differences in the habitat across the sub-regions [51, 50, 48, 52].

### Cross-channel (line) transects

Density and abundance in the cross-channel transects were estimated following distance sampling (DS) methods [38, 53]. Exploratory analyses were performed to assess appropriate truncation distances and to evaluate whether binning the data into pre-specified distance intervals would improve the fit of detection probability models. To model the detection function, data were truncated at 300 m. Half-normal and hazard rate models were considered as the key function. Model selection was conducted using Conventional Distance Sampling (CDS) [38] and Multiple Covariate Distance Sampling (MCDS) [53], starting with simple models and including group size (gs) and platform (pt) (bow and stern) at a time. Model selection was performed using the Akaike’s Information Criterion (AIC) corrected for small sample sizes.

Analyses for line transect data were performed according to survey stratification for habitat types (river channel–the center of the river, dam reservoirs), and by sub-regions. Density was estimated as follows:

$$D_{ij}=\frac{n_{ij}\;E_{ij}\;f(0)}{2L_{ij}\;g(0)}$$ (1)

where, *n*_*ij*_ is the number of groups sighted in habitat *i* and stratum *j*, *E*_*ij*_ is the estimated mean group size for the population in habitat *i* and stratum *j*, *f*(0) is the sighting probability density at zero perpendicular distance (or the inverse of the effective half strip width [ESW]), *L*_*ij*_ is the total transect length in habitat *i* and stratum *j*, and *g*(0) the probability of seeing a group on the transect line.

Sightings collected independently by the bow and stern platforms were used to estimate detection probability on the trackline as: *g*(0) = (1−*q*^2^) [10]. The probability of a group on the transect line being missed by the first platform (bow) given it was seen at the second platform (stern) is *q*. Thus, *g*(0) can be estimated from *g*(0) = (1−(*n*_01_/*n*_1_)^2^), where *n*_1_ is the number of groups sighted from the second platform within 50 m of the transect line, and *n*_01_ the number of these that were missed by the first platform. An estimate of the coefficient of variation (CV) of g(0) estimation also follows Gómez-Salazar et al. [10] methods. Empirical variances, standard errors and CVs for encounter rate, density and abundance were estimated using Distance Sampling methods [38].

### Parallel (strip) transects

To analyze parallel transects, we used a global detection function fitted to line transects of 22 surveys of Amazonian river dolphin (*Inia geoffrensis*) abundance [54] to correct undetected clusters of dolphins following the method proposed by Goméz-Salazar et al. [10]. As such, considering a strip width of 200 meters from the shore, the probability detection for groups sighted between 0 and 50 meters from the trackline (P_1_) is the same of 50–100 m and 100–150 from the shore; and, the probability detection for groups sighted between 50 and 100 m from the trackline (P_2_) is the same of 0–50 m and 150–200 m from the shore. Estimated P_k_ parameters for *Inia* sp. were P_1_ = 0.960 e P_2_ = 0.630 (*shape* = 0.37 (SE = 0.12), *scale* = -2.61 (SE = 0.42)) [54].

The encounter rate was estimated as the mean number of groups sighted by habitat/stratum per km of transect ($\bar{Er}=n_{ij}/l_{ij}/k_{j}$), where *n*_*ij*_ is the number of groups sighted in the habitat type *i* and stratum *j* on transect *l*_*ij*_, and *k*_*j*_ is the number of transects on the stratum *j*. Density in strip transects was estimated by means of stratification for the habitat types river margin, island margin, and channel, also adopting the same post-stratification applied in line transects, as follows:

$$D_{ij}=\frac{E_{ij}\;[\frac{n_{0-50}}{P_{2}}+\frac{n_{50-100}}{P_{1}}+\frac{n_{100-150}}{P_{1}}+\frac{n_{150-200}}{P_{2}}]}{WL_{ij}g(0)}$$ (2)

where, *D*_*ij*_ is the estimated density in the habitat type *i* and stratum *j*, *E*_*ij*_ is the estimated group size for the population in habitat type *i* and stratum *j*, *L*_*ij*_ is the total length of the parallel transects conducted in that habitat *i* and stratum *j*, and *W* is the strip width (200 m).

### Population size and variances for strip and line transects

We obtained abundance by habitat type and stratum through:

$$N_{ij}=D_{ij}\;A_{ij}$$ (3)

where *A*_*ij*_ corresponds to the study area in km^2^ in the habitat type *i* by each stratum *j*, calculated using satellite images as close as possible to the survey time conduction. The satellite images of the area were imported to QGIS open source software version 3.8.1 Zanzibar [54], where polygons for each of the habitat types in the river system were created to calculate the referring area, based on high resolution images.

Standard errors (SE) and coefficients of variation (CV) were obtained for each habitat type and stratum following Gómez-Salazar et al. [10]. The overall population size (*N*_*t*_) in the whole study area was calculated as the sum of abundance in each habitat type and stratum, and the CV of the total estimate was calculated as:

$$CV(N_{t})=\frac{\sqrt{\sum SE(N_{ij})}}{\sum N_{ij}}$$ (4)

## Results

We surveyed a stretch of 585.9 km of the Tocantins River, 276.6 km of which were in line transects and 309.3 km in strip transects. Search effort carried out in each stratum is shown in Table 2. We sighted 138 groups of Araguaian boto (n = 198 individuals). From the 138 groups, 92 (n = 131 animals) were sighted in the cross-river lines and 46 (n = 67 individuals) in strip transects (Table 2).

**Table 2:** Summary of search effort conducted across the study area by stratum/sub-region in relation to the Tucuruí dam in the Tocantins River in 2014.

Stratum	Area (km^2^)	Line			Strip		
		L (km)	*k*	n	L (km)	*k*	n
Downstream	1169	67.8	34	4	184.8	81	21
Reservoir 1	331	42.1	17	4	-	-	-
Reservoir 2	342	93.93	43	42	-	-	-
Upstream	385	72.7	39	32	124.5	58	25

*k*, number of transects; L, realized effort; n, the overall number of sightings. (-) represents no effort.

**Fig 2:**
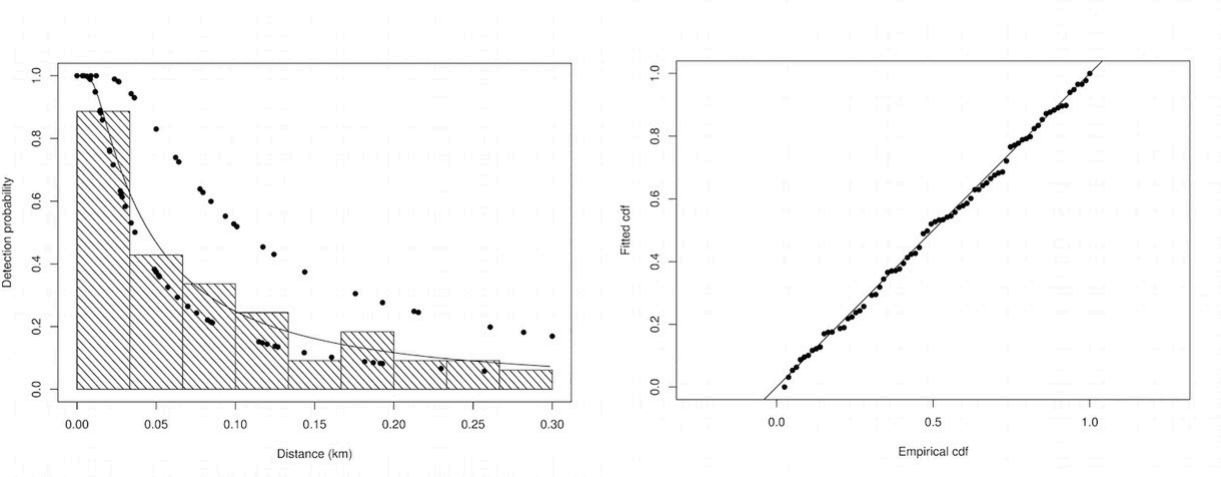
(A) Detection function for the most supported model. (B) Q-Q plot of cumulative distribution function (CDF) of the fitted detection function to the distribution of the data (empirical distribution function or EDF). In (A) line corresponds to the average detection probability (hazard-rate model) and dots the covariate platform P.

From the 92 groups sighted in cross-river transects, 81 (n = 29 for bow platform detections and n = 52 of new stern detections) were used to fit the detection function after accounting for groups detected by both platforms. The number of detections made from the two platforms (duplicates) was low (n = 11 groups, 13%), thus new detections from the stern platform contributed with more than 60% of total detections. The estimated g(0) was 0.659 (CV = 0.262), suggesting a probability of missing dolphins on the trackline of approximately 34%. The hazard-rate model with platform as covariate was the most supported detection probability model according to the AIC (Table 3, Fig 2). This model was then used to estimate density in river channel and reservoir (artificial lake), where cross-river (or cross-channel) transects were surveyed.

**Table 3:** Distance sampling (CDS and MCDS) models for Araguaian boto with Hazard-rate (hr) distributions and covariates.

Modelo	AICc	ΔAICc	P	CV
**hr + *pt***	**343.01**	**0**	**0.26**	**0.22**
hr + *gs* + *pt*	344.11	1.09	0.26	0.26
hr *null*	345.01	2.00	0.28	0.24
hr + *gs*	345.69	2.68	0.26	0.26

Chosen model is highlighted in bold. Supported model within 2 AIC units delimited with dashed lines. P, probability of detection; CV, coefficient of variation.

The overall abundance of the Araguaian boto for non-stratified data was estimated at 2694 animals (95% CI = 1222–4449), versus 2144 animals (95% CI = 1083–3065) in the post-stratified analysis. The initial habitat stratification made prior to the survey, with sampling divided into five habitat types, resulted in high stratum-specific and overall CVs (Table 4). Geographic post-stratification of the data to incorporate the latitudinal and longitudinal trends in density in distinct areas of the study regions (downstream, reservoir 1 and 2, upstream) reduced the CV by approximately 70% (Table 4). The estimated abundance in the reservoirs 1 and 2 contributed to the high variation on final estimates of the post-stratified analysis, since the CIs of both sections were large (95% CI = 67–573 for reservoir 1; 95% CI = 421–1897 for reservoir 2). The most conservative and prudent analysis, taking into account the environmental conditions of the Tucuruí reservoir and sightings distributions, is to consider the estimated abundance based on the lowest number of animals calculated in this habitat. Therefore, we estimate that the total abundance of Araguaian botos in the sampled area is 1083 animals.

**Table 4:** Araguaian boto density and abundance estimates (overall and by habitat/stratum) in the Tocantins River 2014 survey.

Habitat	E(s)	n	L	Er	D	N	CV	A
Without post-stratification								
River margin	1.5	24	205.61	0.1	0.21	195	2.7	927.76
River channel	1.56	33	140.54	0.64	0.94	300	0.4	318.9
Reservoir	1.3	46	136.03	0.33	2.82	1897	0.3	673
Channel	2	5	34.37	0.29	1.86	139	2.12	74.97
Island margin	1.1	10	69.24	0.74	0.7	163	0.36	232.76
TOTAL	1.41	104	585.9	0.42	1.21	2694	1.78	2217.4
With post-stratification								
River margin downstream	1.86	9	106.14	0.07	0.23	30	0.92	133.5
River channel downstream	1.44	4	67.83	0.05	0.02	16	0.67	794.2
Channel downstream	2	3	27.39	0.25	1.68	96	1.27	57.3
Island margin downstream	1.08	6	47.83	0.18	1.24	228	0.50	184.4
Reservoir part 1	1.25	4	42.1	0.09	0.76	244	0.55	331.0
Reservoir part 2	1.43	42	93.93	0.44	3.97	1306	0.39	342.0
River margin upstream	1.40	29	99.47	0.10	0.72	63	0.53	87.4
River channel upstream	1.45	15	72.71	0.38	0.13	30	0.40	231.6
Channel upstream	1	2	6.98	0.10	1.06	19	1.81	17.7
Island margin upstream	1	4	21.41	0.21	2.32	112	0.27	48.4
TOTAL	1.39	104	585.9	0.18	0.75	2144	0.52	2217.4

E(s), group size (number of individuals); n, number of groups sighted; L, realized effort (km); Er, encounter rate (number of groups sighted per km); D, density of animals (ind./km^2^); N, abundance of animals; CV coefficient of variation; A, area of inference (km^2^).

**Fig 3:**
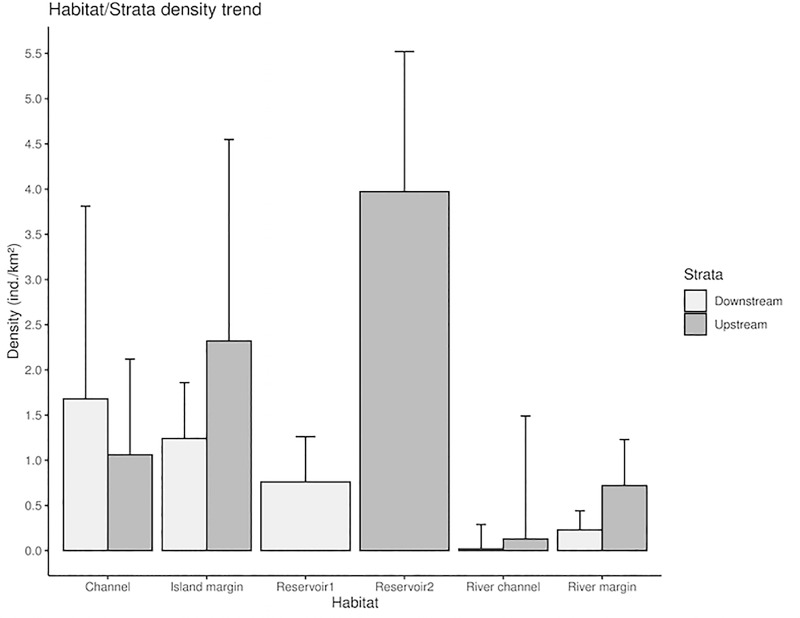
Trend of density for each habitat type surveyed regarding the post-stratification towards the Tucuruí dam in the Tocantins River 2014 survey. Bars represent the standard error (SE) associated.

**Fig 4:**
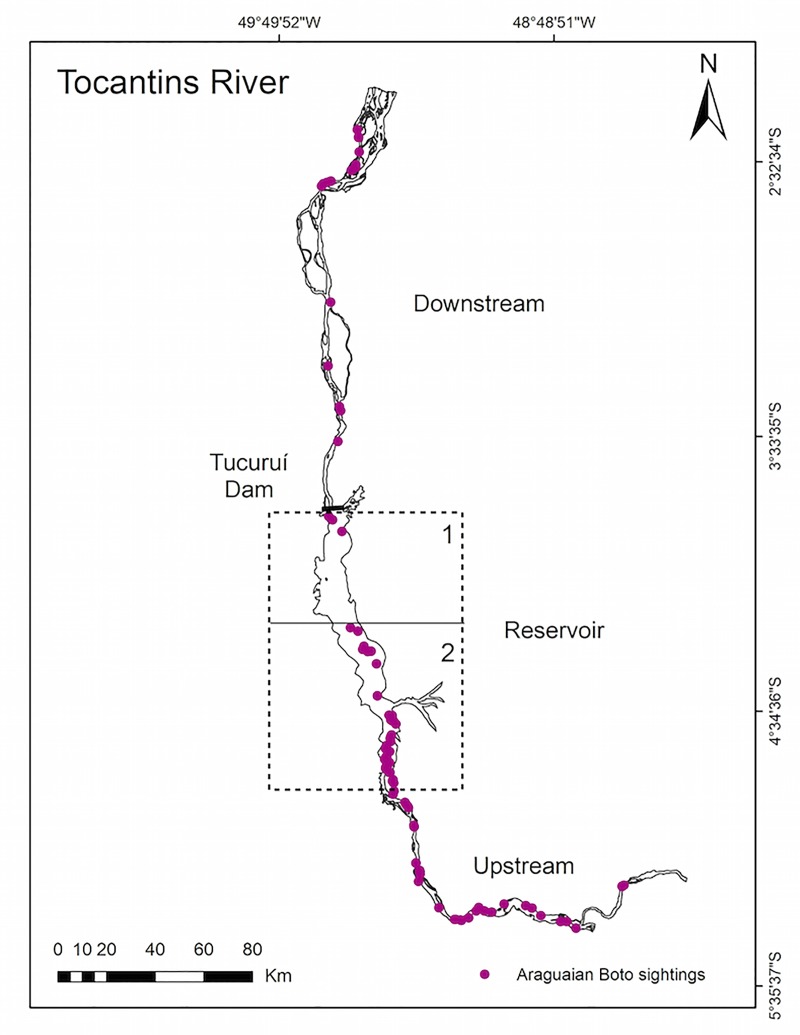
Distribution of Araguaian boto sightings in the Tocantins River 2014 survey. Sightings tend to gradually decrease towards the Tucuruí dam, both in upstream and downstream regions.

Araguaian boto densities decreased from the margin to the center of the river downstream and upstream of the dam but were concentrated in the center within the reservoir. The most dense habitat types were channels and island margins both downstream and upstream of the Tucuruí dam (Table 4). However, lower densities were generally estimated downstream of the Tucuruí dam for all habitat types when compared to upstream, except for channels (Fig 3). Density in the river margin was 68% higher upstream than downstream of the dam, and the resulting abundance estimation was nearly two times larger upstream than downstream for this habitat. Density within the reservoir habitat was highly variable, with sightings decreasing gradually towards the dam (Fig 4). Many sightings were expected to occur in confluences, mainly of the Tocantins and Araguaia rivers, but instead, no dolphins were sighted in this habitat.

## Discussion

This study provides the first estimate of population size of Araguaian botos in the Tocantins River. We found a population of 1083 individuals (CV = 0.52) in the lower-medium course of this human-impacted riverine system. This abundance estimate reflects the results of our post-stratified analysis with the CI lower bound, which we consider as the most robust and reliable way of estimating abundances for the sampled area.

For an equivalent effort employed in the Tapajós River for density and abundance estimation of river dolphins [18], density in all surveyed habitats was substantially smaller in the Tocantins River than the Tapajós River, with river margin and island habitat types presenting the highest differences. Comparison with the Tapajós River is warranted because they are similar in terms of hydro-geomorphology with clear waters, low concentrations of nutrients, ions, and sediments, rocky margins, and presence of rapids [35, 36]. These rivers also have their headwaters in the Central Brazilian Shield and are important waterways for agricultural exports [55]. Due to similar features in the two river basins, one might expect the density and population size of boto to be similar as well. However, the Tapajós River basin is relatively more pristine, whereas the Tocantins Basin is intensively altered by several long-term human activities (e.g., large cities, farms, boat traffic, fishing and agricultural exploitation, hydroelectric dams, and mining), which is likely related to the small population size.

Boto density in the river margin habitat is similar upstream of the Tucuruí dam in the Tocantins River (0.72 ind./km^2^, CV = 0.53) and in the Tapajós River (0.88 ind./km^2^, CV = 0.32). However, the density downstream of the dam in the Tocantins river is lower (0.23 ind./km^2^, CV = 0.92). In the Tapajós River, the highest densities of boto were recorded in island margins (5.7 ind./km^2^, CV = 0.62) in the lower course of the basin, where this habitat is more conspicuous, similarly to the Tocantins River. However, in the Tocantins River we found the highest density along island margins upstream the Tucuruí dam (2.32 ind./km^2^, CV = 0.27), in the middle course of the river, where this habitat is less available compared to the lower course, but may be less altered by Tucuruí dam.

The estimated survey-specific detection probability on the trackline for the Tocantins River (g(0) = 0.659, CV = 0.26) is quite similar to that found in the Tapajós River (g(0) = 0.648, CV = 0.27, [18]). This means that approximately 30% of the dolphin groups were not detected on the survey trackline. These estimates are considerably lower than the g(0) estimated by Gómez-Salazar et al. [10] (g(0) = 0.947, CV = 0.02), where only nearly 5% of dolphins are missed on the survey trackline. Gómez-Salazar et al. [10] have estimated the g(0) based on seven abundance surveys for river dolphins in the Orinoco and Amazon basins. The Gómez-Salazar et al. [10] g(0) was estimated to be used as a global detection probability in Amazonian river dolphins abundance estimation studies. Several Amazon tributaries do not allow proper implementation of line transect designs, and only strip transect surveys can be implemented. In these cases, a global detection probability [10] estimated for other regions is used to correct the assumption of 100% precision on detectability, known to be violated (see [18] for more details). However, for the mainstem of large Amazonian rivers, a global g(0) may smooth important factors that influence detection probabilities (e.g. different observer teams, different platform heights, vessel types, environmental conditions, water level seasonality, and dolphin behavior [40, 43–48]. Thus, we recommend to estimate survey-specific g(0), where transect line design is feasible, to account for variability and to provide more realistic estimates.

The Tucuruí dam, placed in the lower course of the Tocantins River, is likely a factor causing contrasting Araguaian boto density along the river. The impacts of the Tucuruí hydroelectric dam are considerable in terms of habitat transformation, biodiversity and productivity loss, and ecosystem service provisioning [56, 31, 32]. The Tucuruí dam substantially altered hydrological cycles of the Tocantins River both upstream and downstream of the dam. The water flow and levels are directly influenced by the frequency that floodgates are opened. Notably, the dam has dramatically altered the frequency and duration of downstream high and low pulses, as well as the rate and frequency of water condition changes [57]. These changes in hydrology, in addition to changes in water quality, are typically detrimental to downstream biota and biodiversity [58–61].

No surveys were conducted in the Tocantins River prior to the construction of the Tucuruí dam. Therefore, it is difficult to quantify the effect of the dam on the Araguaian boto population. However, if an analogy is made with the history of fragmentation and genetic isolation in Ganges and Yangtze river dolphins populations [21, 62], it is conceivable that the Tucuruí dam has modified the distribution and density of the Araguaian boto population along the lower-medium course of the Tocantins River. Similarly to previous studies [39–41, 3, 10, 11, 18, 63], boto densities in the Tocantins River were higher in the channel and island habitat types (Table 3). Nevertheless, our data indicate that these densities are lower downstream rather than upstream (Fig 4). Araguaian boto density was 68% smaller in river margin downstream than upstream, which is another possible indication of the dam impact.

The Tocantins River is wider and presents more islands and smaller channels along its lower reaches than in the portion of the river upstream of the Tucuruí dam. Therefore, differences in density might also occur due to the relationship between area and probability of detection. Nevertheless, the increased availability of these habitats downstream of the dam does not modify the pattern demonstrated by the downstream pattern data.

The river margin is an important habitat for botos [3, 40]. Dolphin prey typically migrate along the margins, where productivity is higher because of greater concentration of nutrients [35, 64–66]. The Tucuruí dam may have affected distribution of dolphin prey as result of flow changes and decreased sediment load [67, 68]. Dams change sedimentation patterns and cause rivers to undergo major changes in morphology. These changes contribute to reduce the availability of river dolphins’ preferred habitats [69], potentially causing a re-distribution of the dolphins.

Post-stratification of the survey data in smaller habitats was essential to identify latitudinal and longitudinal variation in density. The initial stratification across habitats [10] resulted in estimates of density and abundance with higher CVs. Geographic post-stratification considering the sub-regions in relation to the Tucuruí dam (e.g., downstream and upstream) reduced CVs by as much as 70%, increasing the reliability of our results. However, in most instances CVs are still relatively high (>0.30) and new approaches to sample and analyze data for the Araguaian boto, including an increase in survey effort and potentially modified survey design, should be considered in future surveys. Despite that, we show that geographical stratification, in addition to habitat stratification, is a valuable approach to improve abundance estimates of river dolphins and should be attempted in planning future surveys. The substantial reduction in abundance estimated from stratified compared to non-stratified approaches suggests how data can be biased when results are extrapolated to areas where no sightings were made. Geographic stratification can minimize these biases and prevent overestimation.

We observed high spatial heterogeneity of dolphin sightings within the Tucuruí reservoir. Our results indicate that densities decrease as one moves from upstream areas towards the dam. A previous limnological study in the same region of our sampling investigated aspects of the lower and middle Tocantins River [70]. This previous study identified the existence of three sections with different limnological characteristics inside the Tucuruí dam reservoir. Differences were determined as a function of the system’s hydrodynamics and geomorphology with upstream-downstream spatial distribution and density of zooplankton. The density gradient described for the zooplanktonic community is consistent with the gradient observed for the Araguaian boto inside the reservoir. This spatial pattern has been attributed to physical and chemical differences in water circulation within the reservoir, which causes thermal and oxygen stratification and results in largely anoxic bottom layers as one approaches the dam [70], reducing fish diversity.

In addition to altering the habitat along the Tocantins River, the Tucuruí dam was responsible for the first major break in connectivity in the basin, which resulted in the isolation of groups of Araguaian boto in two stretches of the river. The habitat fragmentation likely interrupted gene flow and may have artificially generated subpopulations of Araguaian botos [71, 15]. Further studies should assess the potential for genetic isolation of dolphins upstream and downstream of the dam.

Hrbek et al. [28] proposed that the Araguaian boto only occurs upstream of the Tucuruí dam, however recent findings demonstrate their presence downstream of the dam as far as the Marajó Island, at the entrance of the Amazon River [72]. Notwithstanding, analysis of both nuclear and mitochondrial DNA revealed that there are hybrids between *Inia araguaiaensis* and *Inia geoffrensis* in the waters downstream of the Tucuruí Dam, therefore limits of distribution of the two species remain unknown (T. Hrbek and J. Farias personal comm).

According to Reeves et al. [73], the distribution of botos in the Tocantins River includes the whole extension of the river. In the upper reaches of the Tocantins River (i.e., upstream of our study area) six other small dams exist within with the range of the Araguain boto. Araújo & Wang [74] suggested that the Araguaian boto population is currently fragmented into eight groups in the Tocantins River. It is known that fragmentation decreases genetic diversity and increases inbreeding [75, 21, 17], which can significantly contribute to population declines and ultimately lead to extinctions [76].

Hundreds of hydroelectric dams have been planned throughout the Amazon, including many in the Tocantins-Araguaia Basin [77–79, 20]. Considering those that are either under construction, planned or inventoried, a total of 24 dams overlap with the distribution of both genera of Amazon river dolphins (*Inia* and *Sotalia*) [78, 74, 18]. Of those, almost one half (11) are concentrated in the Tocantins-Araguaia basin, potentially making the Araguaian boto at high risk of impact by dam construction in South America. Planned dams could split the Araguaian boto population into as many as 12 groups in the Tocantins River and would permanently break the connectivity between dolphins in the Tocantins and Araguaia rivers, further isolating smaller sub-groups of river dolphins in this major Brazilian river basin.

Such marked fragmentation is similar to that faced by Indus river dolphins (*Platanista gangetica minor*), whose population was divided into eight groups in a river blocked by 17 dams [80–82, 62]. Habitat transformation, food depletion, and genetic isolation have caused marked declines in this species density and are compromising the survival of the species [83, 62]. Hydropower development in the Tocantins-Araguaia basin must be planned strategically to minimize similar problems for the Araguaian boto. If more dams are to be constructed, it is recommended that future projects are placed in upstream reaches, where botos are absent. This would prevent additional habitat fragmentation and potentially minimize population declines. This recommendation is consistent with studies on other ecological effects of hydropower dams suggesting that upstream dams tend to be less impactful than mainstem dams [84, 85].

Results from the present study emphasizes the need to re-evaluate the model that South American governments are adopting to obtain energy in the Amazon. This is particularly important for the Tocantins-Araguaia river basin, where many dams have been proposed. Further research is necessary to better assess distribution, density, habitat use and trends in abundance of the Araguaian boto in the Tocantins and Araguaia rivers. This dolphin is under major threats and conservation actions are required to prevent it from having the same fate as that of the Yangtze, Ganges and Indus river dolphins.

## Supporting information

S1 Data (XLSX)
